# Overcoming Access Challenges to Treat Arrhythmias in Patients with Congenital Heart Disease Using Robotic Magnetic-Guided Catheter Ablation

**DOI:** 10.3390/jcm13185432

**Published:** 2024-09-13

**Authors:** Paul Khairy, Katia Dyrda, Blandine Mondésert, Martin Aguilar, Marc Dubuc, Julia Cadrin-Tourigny, Peter G. Guerra, Alexandre Raymond-Paquin, Léna Rivard, Rafik Tadros, Mario Talajic, Bernard Thibault, Laurent Macle, Denis Roy

**Affiliations:** Electrophysiology Service and Adult Congenital Heart Center, Montreal Heart Institute, Université de Montréal, Montreal, QC H1T 1C8, Canada

**Keywords:** congenital heart disease, arrhythmias, catheter ablation, robotics, magnetic guidance

## Abstract

The prevalence of congenital heart disease (CHD) has surged in recent decades, owing to a substantial reduction in mortality. As individuals with CHD age, they become increasingly susceptible to late complications including arrhythmias. These arrhythmias often arise decades after surgical intervention and significantly impact quality of life, hospitalizations, and mortality. Catheter ablation has gained widespread acceptance as a critical intervention for managing arrhythmias in patients with CHD. However, anatomical and physiological features unique to this population pose challenges to standard manual ablation procedures, potentially impacting safety and efficacy. Robotic magnetic-guided navigation (RMN) has emerged as a technological solution to address these challenges. By utilizing soft and flexible catheters equipped with magnets at their tips, RMN enables robotic steering and orientation of catheters in three-dimensional space. This technology overcomes obstacles such as distorted vascular pathways and complex post-surgical reconstructions to facilitate access to target chambers and improve maneuverability within the heart. In this review, we present an overview of the safety and efficacy evidence for RMN-guided catheter ablation in CHD patients and highlight potential advantages. Additionally, we provide a detailed case presentation illustrating the practical application of RMN technology in this population. Although the literature on RMN-guided ablation in patients with CHD remains limited, it has shown promise in achieving successful outcomes, particularly in cases where manual ablation failed or was deemed non-feasible. Further validation through large-scale prospective studies is necessary to fully ascertain the benefits of RMN technology in this patient population.

## 1. Introduction

The prevalence of congenital heart disease (CHD) continues to rise, owing to substantial improvements in survival over the past four decades [1]. As individuals afflicted with CHD age, they become increasingly susceptible to late complications stemming from their underlying condition, as well as hemodynamic and post-operative sequelae. Arrhythmias frequently manifest decades after surgical interventions, impacting quality of life, morbidity, and mortality [2,3,4]. Catheter ablation stands as a pivotal intervention for managing arrhythmias in patients with CHD [5,6]. Nevertheless, the unique anatomical and physiological features encountered in this complex population render standard manual catheter ablation procedures more challenging. Safety and efficacy can potentially be compromised by numerous hurdles. These include challenging access to the chamber of interest due to distorted, obstructed, or disconnected vascular pathways; difficulties in catheter maneuverability within the heart due to congenital defects, post-surgical reconstructions, or the presence of intracardiac devices such as prosthetic valves or septal occluders; deviations from conventional anatomical landmarks for orientation, such as the coronary sinus ostium or bundle of His; and impediments to the delivery of effective ablation lesions, such as prosthetic material coverage over targeted tissue, division of tissue between two post-surgical chambers, and other inherent obstacles hindering adequate catheter stability and contact.

Robotic magnetic-guided navigation (RMN) has emerged as a technological solution that addresses several of these hindrances. Utilizing malleable catheters equipped with magnets at their tips, RMN facilitates their steering and orientation in three dimensions through the manipulation of a pair of large rare earth magnets. Herein, we provide a synopsis of the evidence on the safety and efficacy of RMN-guided catheter ablation in patients with CHD and describe pertinent advantages of this technology. We then explore a detailed case presentation to provide a practical overview of the various steps involved in applying this technology to patients with CHD.

## 2. Systematic Review with Pooled Analyses of RMN in CHD

A recently published systematic review with pooled analyses that included 24 studies on a total of 167 patients with CHD assessed the safety and efficacy of RMN-guided catheter ablation [7]. Characteristics of patients and procedures are outlined in Table 1. The complexity of CHD was categorized as simple in 16%, moderate in 19%, complex in 64%, and undetermined in 1%. The previously described classification system for complexity of CHD is summarized in Table 2 [8]. Patients ranged in age from 13 to 63 years, with a mean age of 36 years. Males constituted 55% of the cohort. A total of 202 procedures were performed in the 167 patients, resulting in an average of 1.2 procedures per patient.

### 2.1. Characteristics of the Arrhythmias and Catheter Trajectories

Among studies included in the systematic review, ablation with RMN was attempted in a total of 260 arrhythmias, averaging 1.6 arrhythmias per patient [7]. The majority (83%, N = 215) of targeted arrhythmias were incisional and focal atrial tachycardias. Atrial fibrillation, atrioventricular (AV) reentrant tachycardia, twin AV node reentrant tachycardia, AV nodal reentrant tachycardia, and ventricular arrhythmias each accounted for 3 to 4% of all targeted arrhythmias.

The primary approach for most procedures involved femoral venous access, while the second most common trajectory utilized a retrograde aortic route through a femoral artery. Reasons cited for opting for a retrograde aortic approach included (i) circumventing a transbaffle puncture to reach the pulmonary venous atrium in patients with atrial switch surgery; (ii) avoiding a transseptal puncture to access the left atrium following percutaneous closure of an atrial septal defect (ASD) or in the setting of congenital absence of the inferior vena cava (IVC); and (iii) bypassing a transconduit puncture to reach the pulmonary venous atrium (PVA) in patients with single ventricle physiology after a total cavopulmonary connection Fontan procedure. In cases of congenital absence of the IVC or acquired obstruction of femoral veins, a superior venous approach via the right or left jugular or subclavian veins was undertaken. Additional catheter access routes encompassed transbaffle or transeptal punctures, passage through the hemi-azygos continuation of an interrupted IVC, use of the brachial artery or vein, and transhepatic approaches [7].

### 2.2. Efficacy

**Figure 1:**
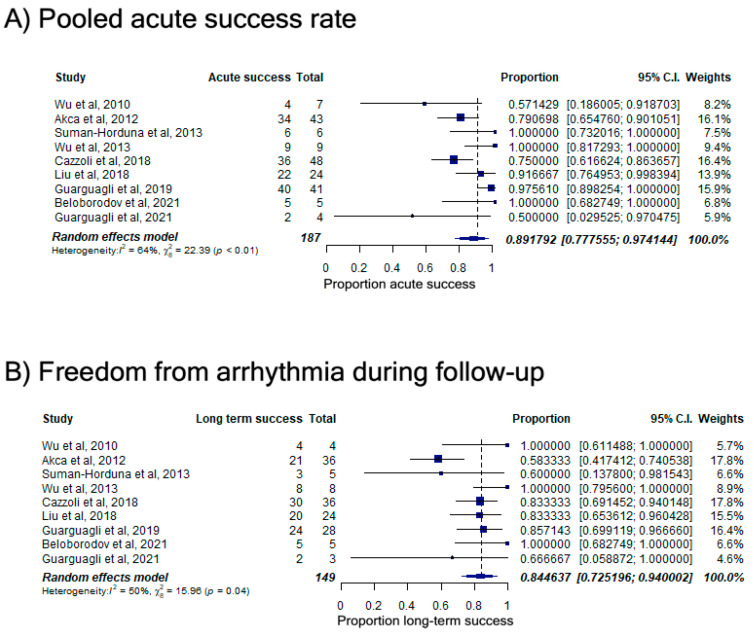
Acute success rate (**A**) and freedom from arrhythmias during follow-up (**B**) by pooled analyses. Shown are the pooled analyses using random effects models for (**A**) the acute success rate and (**B**) recurrence-free survival-associated RMN-guided catheter ablation in patients with CHD. CI denotes confidence interval. Reproduced with permission from Vo C et al. [7].

In the systematic review, various definitions were employed to determine acute success, contingent upon the targeted arrhythmias. Illustrated in Figure 1A, the aggregated acute success rate utilizing a random effects model stood at 89.2% [95% confidence interval (CI; 77.8%; 97.4%)]. Although the definition of long-term success was not uniform across studies, the combined long-term success reached 84.5% [95% CI (72.5%; 94.0%)] over an average follow-up period of 24 months (Figure 1B). While a direct comparative analysis was beyond the scope of the systematic review, the acute success rate achieved with RMN-guided ablation surpassed previously reported rates of acute success obtained by manual catheter ablation for various arrhythmias in patients with CHD (80–81%) [6,9]. The introduction of irrigated-tip RMN-guided ablation catheters in the early 2010s is believed to have substantially improved efficacy by facilitating the creation of deeper myocardial lesions, a particularly relevant advancement for patients with CHD, who typically present with thickened chamber walls. Exclusion of studies employing the earlier non-irrigated RMN-guided radiofrequency ablation catheter resulted in an acute success rate surpassing 93%. Caution is advised in interpreting longer-term success rates due to the absence of standardized definitions, variations in ablation technologies (e.g., irrigated versus non-irrigated radiofrequency energy), and differences in follow-up durations.

### 2.3. Safety

Although there was variability in the provided definitions and level of detail regarding complications among studies included in the systematic review, at least one complication was documented in 7 of 202 procedures, resulting in a procedural complication rate of 3.5%. Among these, 7 of 8 complications were vascular in nature, comprising 5 cases of groin hematomas and 2 instances of pseudoaneurysms, with one patient experiencing both a groin hematoma and pseudoaneurysm. The sole remaining complication entailed a hemothorax resulting from the insertion of a central line for anesthesia administration. Importantly, there were no documented complications directly associated with the RMN technology, such as cardiac perforation, damage to the aortic valve, AV block, or stroke. Rare complications reported in other patient cohorts without CHD undergoing RMN-guided ablation have included pericardial tamponade [10] and catheter knotting or entrapment [11].

### 2.4. General Inferences

As evidenced by the systematic review, the utilization of RMN in patients with CHD has been explored in limited published studies. Nevertheless, the findings indicate its safe and effective application in managing various arrhythmias across a broad spectrum of disease severity, encompassing over 100 patients with complex CHD. No safety issues have been reported, and initial success rates are promising. Notably, specific patient subgroups within the CHD population appear to benefit most from this technology, including those with (1) venous anomalies such as congenital absence or interruption of the IVC or bilateral femoral venous occlusion; (2) specific intracardiac anatomies, such as atrial baffles and intracardiac prostheses; and (3) challenging-to-access pulmonary venous chambers (e.g., total cavopulmonary connection Fontan and closure of ASDs).

## 3. Attractive Features of RMN-Guided Ablation in CHD

Magnetic-guided catheters exhibit flexibility and versatility, which render them capable of navigating through multiple angles with precision. Their pliability makes them less susceptible to mechanical constraints, allowing the catheters to be steered through complex trajectories. Control of the magnetic field’s vector and catheter advancement is achieved through intuitive mouse and scroll wheel movements. Since the magnetic field vector orients the tip of the catheter, maneuverability is not hindered by successive loops or sharp-angle turns that may have been required to reach the cardiac chamber of interest.

RMN-guided ablation catheters are not equipped with sensors that provide exact contact force measurements. However, they incorporate an e-Contact module that utilizes impedance measurements, tracks the cardiac-induced motion of the catheter, and analyzes magnetic torque data to generate a contact evaluation depicted visually in a semi-quantitative manner as a starburst pattern at the tip of the catheter. An advantage of RMN is its ability to maintain constant contact with the beating heart [12]. Unlike the stiffer pull-wire catheters used in manual ablation, the flexible magnetic catheter behaves akin to a shock absorber, ensuring consistent contact and stability throughout the cardiac cycle. Ablation lesions produced by RMN exhibit greater consistency and density compared to manual methods, as demonstrated on gel models [12].

We previously demonstrated that the catheter is associated with minimal deviation in contact force, indicative of consistent performance [13]. Contact force assessment revealed an average force of 6.1 g without a sheath, increasing to 20.4 g with a sheath positioned at the chamber entrance. The compliant catheter buckles with attempts to increase contact force beyond 22 g. This likely contributes to its excellent safety profile, with no reported case of mechanical cardiac perforation. Other advantages include a reduction in fluoroscopy exposure [7], greater operator comfort that is much appreciated during lengthy procedures, and sophisticated built-in features such as “ablation history” that visually depicts ablation lesions in three-dimensional space, reflects power output and duration, and offers insights into lesion density, width, and potential gaps.

## 4. Cost Considerations

The cost-effectiveness of RMN-guided ablation in CHD is challenging to assess due to several factors. While RMN systems involve significant upfront costs, including equipment expenses, disposables, installation, and maintenance fees, these costs must be weighed against potential long-term benefits. RMN improves catheter access and has the potential to enhance precision and reduce procedural complications, which could lead to fewer repeat interventions and improved overall outcomes. However, there are no empirical data specifically addressing the cost-effectiveness of RMN in CHD. In cases where manual catheter ablation procedures are infeasible or fail, the balance would favor an initial RMN-based strategy. For the larger CHD population, it remains to be determined whether the advantages of RMN compared to manual ablation ultimately offset its higher initial financial outlay. This hypothesis requires further investigation to provide a comprehensive economic evaluation.

## 5. Case Illustration

To illustrate the utility of RMN-guided ablation in CHD, a case example is provided that describes the various steps involved, including registration; importing, segmenting, and annotating 3D images; electroanatomic mapping; merging electroanatomic maps with 3D imaging; maneuvering the catheter across complex trajectories; and catheter ablation.

### 5.1. Case History

A 34-year-old woman with complex CHD had recurrent arrhythmias associated with presyncope and heart failure. She was referred for robotic ablation after a failed standard manual procedure. She was born with right atrial isomerism, bilateral superior vena cavae (SVC), two large IVCs draining into the right atrium, asplenia, left-sided liver, unbalanced AV septal defect with a common AV valve, hypoplastic left ventricle, double-outlet right ventricle (DORV) with pulmonary stenosis and mild aortic stenosis, and sinus venosus-type ASD. She had a series of surgical interventions including right and left Glenn shunts, culminating in a modified intra-cardiac tunnel Fontan with an intra-atrial Goretex tube that directed flow from both IVCs to the main pulmonary artery (PA). An epicardial pacemaker was implanted for sinus node dysfunction.

**Figure 2:**
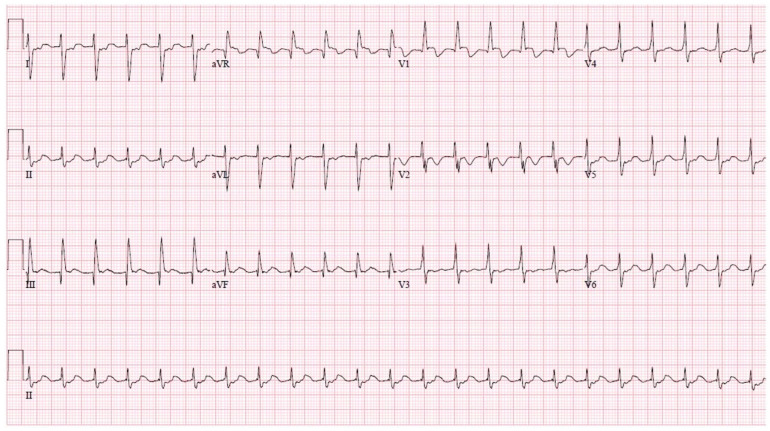
A 12-lead electrocardiogram of the presenting arrhythmia. The clinical tachycardia is captured at a rate of 135 bpm. The pattern of right ventricular hypertrophy is consistent with a single ventricle of right ventricular morphology.

The presenting electrocardiogram during tachycardia is shown in Figure 2. A slow atrial arrhythmia with 1:1 AV conduction was suspected. The electrophysiological procedure was performed under local anesthesia with conscious sedation. An 8-French sheath was introduced in the right femoral artery and 2 venous sheaths (8-French; 6-French) in the right femoral vein under ultrasound guidance. An activated clotting time of 350 s was maintained throughout the procedure. Initial mapping of the Fontan pathway revealed far-field atrial electrograms at the junction with the left PA (LPA). There, a reference decapolar catheter was positioned.

### 5.2. Overview of the RMN System and Registration

**Figure 3:**
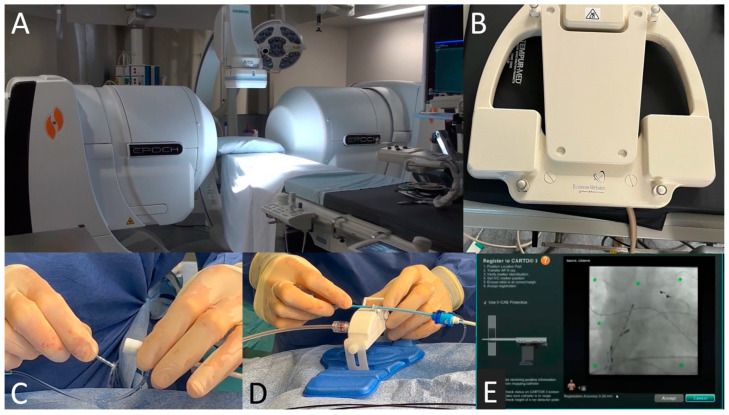
The RMN-guided catheter ablation system. Shown in Panel (**A**) are two large rare earth magnets on each side of the fluoroscopy table. The location pad displayed in Panel (**B**) is positioned under the table and allows integration of magnetic guidance with 3D electroanatomic mapping and fluoroscopy. The irrigated radiofrequency magnetic-guided ablation catheter is flushed before insertion into a sheath (**C**) and then connected to a motor (**D**) to allow remote control. In Panel (**E**), the location pad is centered such that 6 references markers (in green) are seen within an a posteroanterior fluoroscopy view.

Shown in Figure 3A are large (1.8-ton) rare earth neodymium-iron-boron magnets positioned on each side of the fluoroscopy table (Epoch platform, Stereotaxis, St. Louis, MO, USA). They create a relatively uniform magnetic field (~15 cm in diameter) around that patient’s thorax consisting of 0.1 Tesla in any direction. The location pad shown in Figure 3B is positioned underneath the fluoroscopy table and contains a registration plate module. This serves to align the 3D-electroanatomic mapping system (CARTO 3, Biosense Webster, Johnson & Johnson, South Diamond Bar, CA, USA) with conventional fluoroscopy and the RMN platform. Doing so requires centering 6 reference markers (green dots in Figure 3E) in a standard posteroanterior fluoroscopy view and positioning the RMN-guided ablation catheter (Navistar RMT Thermocool, Biosense Webster) within the confines of the markers. In Figure 3C, the RMN-guided irrigated radiofrequency ablation catheter is flushed before inserting it into a long sheath. It is subsequently connected to a motor (Figure 3D; QuikCAS Cardiodrive, Stereotaxis) that allows the catheter to be advanced and retracted remotely using the scroll wheel of a mouse.

### 5.3. Importing, Segmenting, Annotating 3D Images

**Figure 4:**
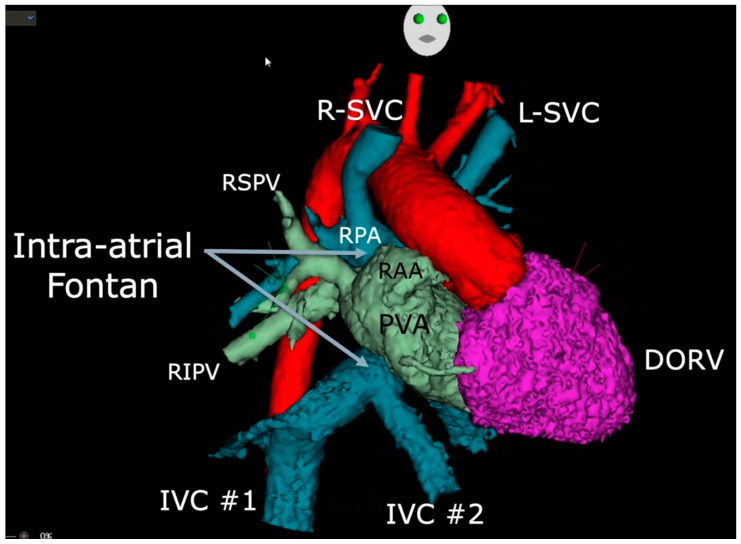
CT scan images segmented and imported into a 3D-electroanatomic mapping system. R-SVC and L-SVC denote right and left superior vena cavae; RSPV, right superior pulmonary vein; RPA, right pulmonary artery; RAA, right atrial appendage; RIPV, right inferior pulmonary vein; PVA, pulmonary venous atrium; DORV, double-outlet right ventricle; IVC, inferior vena cava.

A crucial aspect of the procedure involves the acquisition, segmentation, and annotation of three-dimensional images, usually obtained from preprocedural computed tomography (CT) scans or cardiac magnetic resonance (CMR) imaging. This facilitates the visualization of anatomic intricacies and serves as a navigational guide for directing the RMN-guided ablation catheter. Figure 4 presents a static view of the patient’s cardiac anatomy that was imported into the 3D-electroanatomic system and semi-automatically segmented utilizing the CARTOSEG CT module (Biosense Webster). A three-dimensional rotational view is provided in Supplementary Video S1.

### 5.4. Electroanatomic Mapping

**Figure 5:**
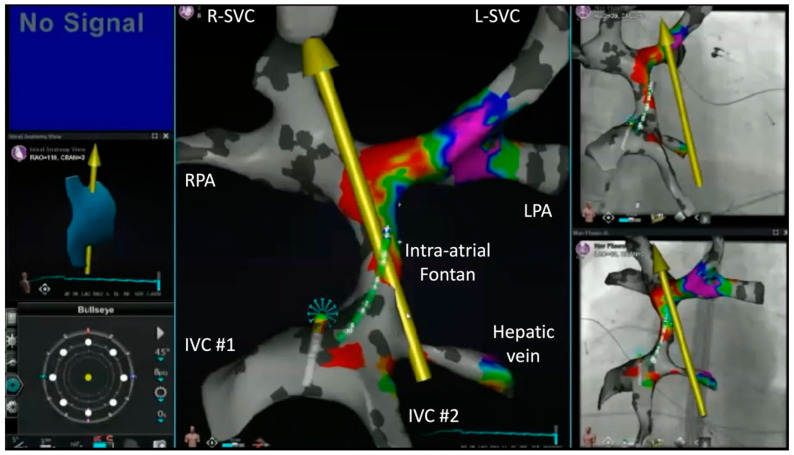
Electroanatomic mapping of the systemic venous atrium. R-SVC and L-SVC denote right and left superior vena cavae; RPA and LPA, right and left pulmonary arteries; IVC, inferior vena cava. The yellow arrow designates the direction of the magnetic field and the starburst at the tip of the ablation catheter indicates good tissue contact.

As shown in Figure 5, 3D-electroanatomic mapping is typically initially performed in the more easily accessible chamber. This serves to determine whether or not a critical component of the arrhythmia substrate is located within this chamber. Voltage mapping may also facilitate the selection of an optimal site for the placement of a stable reference catheter. Options may be limited in the context of synthetic material (such as the Goretex conduit in this patient) or extensive scarring. Moreover, acquired images can serve as a foundation for merging with CT scan or MRI images, facilitating navigation to more challenging-to-access chambers. The yellow arrow in Figure 5 indicates the axis of the magnetic field vector that could be guided in any direction in three-dimensional space by changing the orientation of the magnets relative to one another. The starburst projected around the tip of the RMN-guided ablation catheter indicates good tissue contact. A decapolar reference catheter is seen within the intra-atrial Fontan conduit. The right-sided panels of Figure 5 display the electroanatomic map overlayed onto static fluoroscopy images in right and left anterior oblique views.

### 5.5. Merging Electroanatomic Mapping with 3D Imaging

**Figure 6:**
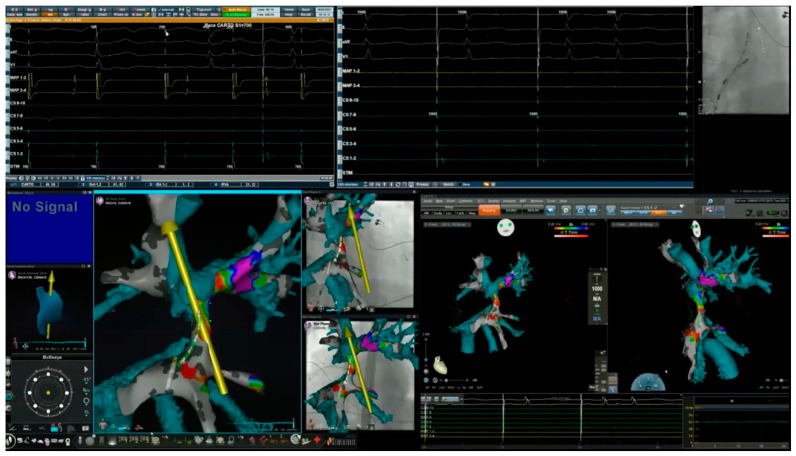
Merging of electroanatomic mapping and CT scan images of the systemic venous atrium in multiple views. Shown is a standard screen view from the control room during an ablation procedure, with the upper panels capturing snapshot (left) and real-time (right) electrogram tracings. The bottom panels display the operator view of CT scan and 3D mapping images (left) during the merging process, overlaying of these images on orthogonal fluoroscopic views (middle), and views from the 3D electroanatomic mapping system. The yellow arrows indicate the direction of the magnetic field.

Figure 6 provides a screenshot of the operator’s view from the workstation during the process of merging electroanatomic mapping and CT scan images of the Fontan conduit, SVC, IVC, and PAs. The process involves aligning the anatomical features identified in the imaging data with the corresponding locations on the electroanatomic map in multiple views. By registering the spatial coordinates of the two datasets, a unified representation of the patient’s cardiac anatomy and superimposed electrical activation patterns is generated. The merged dataset serves as a valuable tool for guiding catheter navigation. The overlaying of the electroanatomic map onto the 3D images allows arrhythmias to be precisely localized and targeted for ablation. The merging process can be further refined as additional electroanatomic mapping data become available. In particular, a fast anatomic map of the aorta can help maximize the accuracy of the merged dataset.

### 5.6. Retrograde Aortic Access

**Figure 7:**
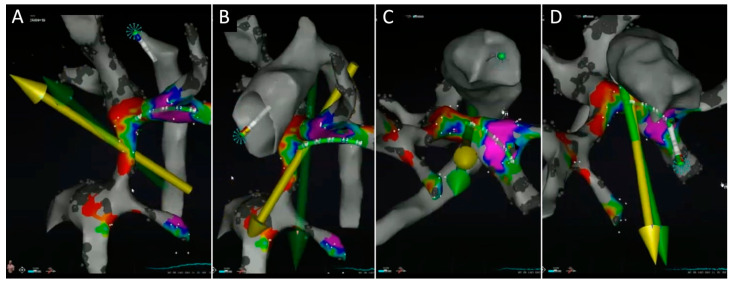
RMN-guided retrograde aortic access.

**Figure 8:**
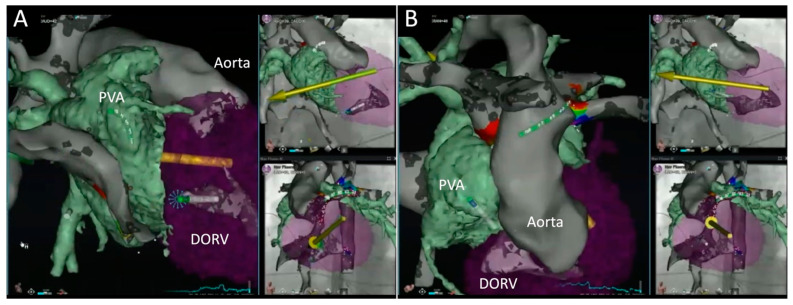
Crossing the common AV valve of the double-outlet right ventricle (DORV) to enter the pulmonary venous atrium (PVA). The green arrows represent the direction of the magnetic field set by the operator whereas the yellow arrows indicate the actual direction of the magnetic field, with a brief time lag between the two.

After excluding the possibility that the arrhythmia was located in the systemic venous atrium, the pulmonary venous atrium (PVA) was accessed using a retrograde aortic approach. This involved crossing the aortic valve, entering the DORV, and traversing the common AV valve to reach the PVA. Figure 7 follows the ablation catheter’s trajectory as it courses up the descending aorta (Figure 7A), reaches the ascending aorta (Figure 7B), delineates the aortic root (Figure 7C), and crosses the aortic valve to enter the DORV (Figure 7D). To facilitate entry into the ventricle, the image was tilted so as to obtain a clock-face view of the aortic valve. This allows the catheter to be oriented toward the center of aortic valve (Figure 7C), which could be readily crossed during systole without the need to prolapse the catheter and create a large “J” loop. The merged CT scan images were displayed once the catheter reached the DORV (Figure 8) to assist in guiding it toward the PVA. As shown in Figure 8A, the ablation catheter was directed toward the base of the DORV near the common AV valve annulus. As shown in Figure 8B, the common AV valve was crossed and the catheter is seen within the PVA.

### 5.7. Mapping and Ablation in the Pulmonary Venous Atrium

**Figure 9:**
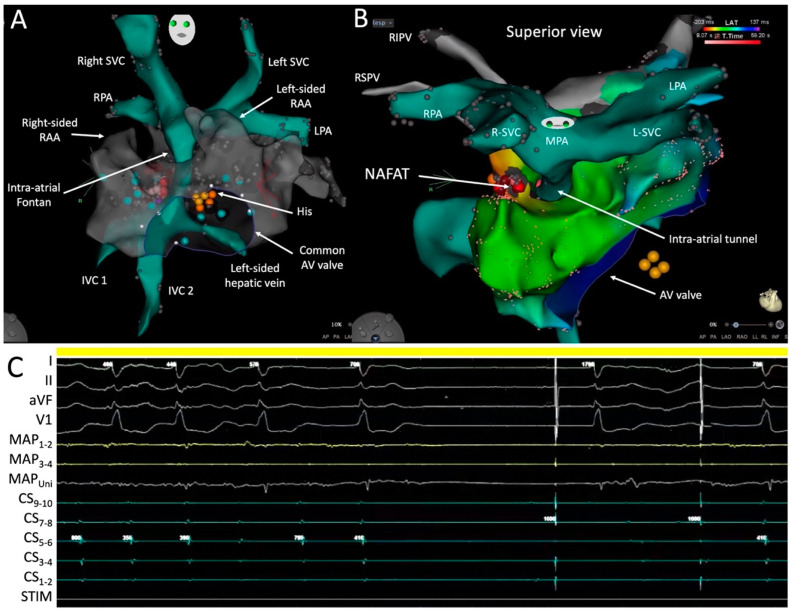
Mapping and ablation in the pulmonary venous atrium. Shown in Panels (**A**,**B**) are 3D views of electroanatomic maps. R- and L-SVC denote right and left superior vena cavae; RPA and LPA, right and left pulmonary arteries; RAA, right atrial appendage; AV, atrioventricular; IVC, inferior vena cava; RIPV, right inferior pulmonary vein; RSPV, right superior pulmonary vein; MPA, main pulmonary artery; NAFAT, non-automatic focal atrial tachycardia. Shown in the tracing in Panel (**C**) are surface ECG leads I, II, aVF, and V1; intracardiac recordings from the distal (MAP_1-2_) to proximal (MAP_3-4_) and unipolar (MAP_Uni_) mapping catheter; and the proximal (CS_9-10_) to distal (CS_1-2_) coronary sinus catheter. STIM denotes the stimulation channel.

Once in the PVA, a fast anatomical map was created to provide a geometric shell and localize the AV conduction system. This step is vital in patients who have displaced or difficult-to-predict locations of the AV node to avoid inadvertent damage during ablation. Figure 9A shows the position of the His signal recorded along the superior border of the common AV valve. The relationship between the systemic venous atrium and PVA could be appreciated. The intra-atrial conduit is surrounded by the PVA and pierces its roof before anastomosing with the main PA (MPA).

During single atrial extra-stimulus testing at a drive train of 600 ms, the clinical tachycardia, which had a cycle length of 440 ms, was easily induced. Activation and entrainment mapping revealed a non-automatic focal atrial tachycardia (NAFAT) on the roof of the PVA, adjacent and to the right of the intra-atrial tunnel alongside scar tissue (Figure 9B). At this site, catheter ablation resulted in acceleration of the tachycardia followed by slight slowing before termination (Figure 9C). A propagation map of the tachycardia is shown in Supplementary Video S2. Tachycardia was no longer inducible post ablation and the patient has remained arrhythmia-free since. Ideal procedural endpoints include termination of all focal atrial tachycardias during ablation, demonstration of bidirectional block across linear ablation lesions if relevant, and non-inducibility of any atrial tachycardia.

## 6. Future Perspectives

The challenges encountered during catheter ablation in patients with CHD are multifaceted, encompassing the presence of multiple arrhythmias, complex substrates, limitations in accessing the target chamber, and obstacles hindering the formation of effective ablation lesions. These impediments may reflect anatomical constraints or limitations in catheter reach, maneuverability, and/or stability. In this context, RMN technology has emerged as a promising solution. Its theoretical advantages align well with the challenges posed by the CHD population.

Although the literature on RMN-guided ablation in CHD patients remains limited, it has proven to be an essential tool for achieving successful outcomes in selected cases for whom manual ablation was attempted and failed or was deemed non-feasible. In our experience, these patients generally fall into one of the following three categories: (a) inadequate vascular access for manual ablation (e.g., obstructed femoral veins or interrupted/obstructed IVC); (b) successful access to the chamber of interest using a manual approach but inability to reach the arrhythmia target (e.g., a trans-septal puncture that orients the catheter away from the site of interest or an excessively large chamber that compromises catheter stability); and (c) inability to reach the chamber of interest manually despite adequate vascular access (e.g., conduit that cannot be punctured due to safety concerns or excessive calcification). Diverse forms of arrhythmias have been effectively managed using the RMN-based approach, even among the most complex anatomies. It is noteworthy that no specific safety concerns related to RMN-guided ablation were identified in this patient population. Further validation of these promising findings is warranted through large-scale multicenter and prospective studies.

## Figures and Tables

**Table 1:** Patient and procedural characteristics.

Characteristic	Summary Statistic	Denominator
Number of patients, N	167	167
Age, years (mean (range))	36.4 (13–64)	158
Female sex, N (%)	70 (44.6)	157
Congenital heart disease complexity, N (%) Simple Moderate Complex	27 (16.4) 32 (19.4) 106 (64.2)	165
Number of procedures, N Number of procedures per patient	202 1.21	202
Number of targeted arrhythmias, N	260	260
Type of arrhythmia targeted, N (%) Incisional or focal atrial tachycardia ○ Intra-atrial reentrant tachycardia ○ Atrial tachycardia, not further specified ○ Non-automatic focal atrial tachycardia ○ Atrial flutter Atrial fibrillation Atrioventricular reentrant tachycardia Twin AV node reentrant tachycardia Ventricular tachycardia	215 (82.7) 147 (56.5) 58 (22.3) 7 (2.7) 3 (1.2) 11 (4.2) 9 (3.5) 9 (3.5) 8 (3.1)	260
Average number of targeted arrhythmias per procedure, N	1.29	202
Average number of targeted arrhythmias per patient, N	1.56	167
Number of trans-septal/trans-baffle punctures, N (%)	10 (5.0)	202

Reproduced with permission from Vo C et al. [7].

**Table 2:** Classification of congenital heart disease complexity.

Complexity	Type of Congenital Heart Disease
**Simple**	*Native disease* Isolated congenital aortic valve disease Isolated congenital mitral valve disease (except parachute valve, cleft leaflet) Small atrial septal defect Isolated small ventricular septal defect (no associated lesions) Mild pulmonary stenosis Small patent ductus arteriosus *Repaired conditions* Previously ligated or occluded ductus arteriosus Repaired secundum or sinus venosus atrial septal defect without residua Repaired ventricular septal defect without residua
**Moderate**	Aorto-left ventricular fistulas Anomalous pulmonary venous drainage, partial or total Atrioventricular septal defects, partial or complete Coarctation of the aorta Ebstein anomaly Infundibular right ventricular outflow obstruction of significance Ostium primum atrial septal defect Patent ductus arteriosus, not closed Pulmonary valve regurgitation, moderate to severe Pulmonary valve stenosis, moderate to severe Sinus of Valsalva fistula/aneurysm Sinus venosus atrial septal defect Subvalvular or supravalvular aortic stenosis Tetralogy of Fallot Ventricular septal defect with the following: Absent valve or valves Aortic regurgitation Coarctation of the aorta Mitral disease Right ventricular outflow tract obstruction Straddling tricuspid or mitral valve Subaortic stenosis
**Severe**	Conduits, valved or nonvalved Cyanotic congenital heart disease, all forms Double-outlet ventricle Eisenmenger syndrome Fontan procedure Mitral atresia Single ventricle (also called double inlet or outlet, common, or primitive) Pulmonary atresia, all forms Pulmonary vascular obstructive disease Transposition of the great arteries Tricuspid atresia Truncus arteriosus/hemitruncus Other abnormalities of atrioventricular or ventriculoarterial connection not included above (e.g., crisscross heart, isomerism, heterotaxy syndromes, ventricular inversion)

Reproduced with permission from Khairy P et al. [8].

## Supplementary Materials

The following supporting information can be downloaded at https://www.mdpi.com/article/10.3390/jcm13185432/s1: Video S1: 3D rotational view of the segmented and imported CT scan images of the heart; Video S2: Propagation map of the non-automatic focal atrial tachycardia.
